# Analysis of sesquiterpene hydrocarbons in grape berry exocarp (*Vitis vinifera* L.) using in vivo-labeling and comprehensive two-dimensional gas chromatography–mass spectrometry (GC×GC–MS)

**DOI:** 10.3762/bjoc.15.190

**Published:** 2019-08-14

**Authors:** Philipp P Könen, Matthias Wüst

**Affiliations:** 1 Institute of Nutritional and Food Sciences, Chair of Food Chemistry, University of Bonn, Endenicher Allee 19C, 53115 Bonn, Germany

**Keywords:** biosynthesis, deuterium labeling, germacrene, HS-SPME, terpenes, TOF–MS

## Abstract

Sesquiterpenes are structurally diverse, potent flavoring substances that significantly influence the aroma profile of grapes (*Vitis vinifera* L.) at the time of physiological ripening. To investigate these natural compounds, freshly harvested, ripe berries of the red wine variety Lemberger (*Vitis vinifera* subsp. *vinifera* L.) were analyzed using comprehensive two-dimensional gas chromatography (GC×GC) coupled to a time-of-flight mass spectrometer (TOF–MS) after headspace-solid phase microextraction (HS-SPME). The identification of structurally complex natural compounds, such as sesquiterpenes from fruits and vegetables, is often reported as “tentative”, as authentic standards are not commercially available for most of the analytes. For this reason, feeding experiments (in vivo labeling) were carried out using the stable isotope-labeled precursors [5,5-^2^H_2_]-1-deoxy-ᴅ-xylulose (*d*_2_-DOX) and [6,6,6-^2^H_3_]-(±)-mevalonolactone (*d*_3_-MVL) to clearly identify the volatiles. Based on the recorded mass spectra of the unlabeled and deuterated compounds, mechanisms for sesquiterpene formation in *V. vinifera* could be proposed and already known pathways could be confirmed or disproved. For example, the HS-SPME–GC×GC–TOF–MS measurements of fed sample material showed that the tricyclic sesquiterpene hydrocarbons α-copaene, β-copaene, α-cubebene, β-cubebene and the bicyclic δ-cadinene were biosynthesized via (*S*)-(−)-germacrene D rather than via (*R*)-(+)-germacrene D as intermediate.

## Introduction

The aroma profile of grape berries at the time of physiological ripening is very complex and significantly influenced by potent flavoring substances of isoprenoid origin like mono- and sesquiterpenes [1–2]. Sesquiterpenes form a structurally diverse subgroup of terpenes consisting of three isoprene units [3]. Since the bicyclic sesquiterpene ketone rotundone was identified for the first time in 2008 as a key aromatic substance for the peppery aroma of the red wine variety Shiraz, sesquiterpenes have increasingly been in the focus of wine research [4].

It is known that these natural products are synthesized in *Vitis vinifera* L. via the mevalonate-dependent biosynthesis pathway (MVA) localized in the cytoplasm as well as via the mevalonate-independent 1-deoxy-ᴅ-xylulose 5-phosphate/2-*C*-methyl-ᴅ-erythritol 4-phosphate metabolic pathway (DOXP/MEP) localized in plastids [5]. In the MVA pathway, which was first described in yeast and animals, mevalonic acid (MVA) is formed from acetyl-CoA and converted into the products isopentenyl pyrophosphate (IPP) and dimethylallyl pyrophosphate (DMAPP), which can further react to farnesyl pyrophosphate (FPP, C_15_) [6]. It was not until the end of the 1980s that the alternative DOXP/MEP pathway, which also leads to the formation of DMAPP and IPP, was discovered in a study focused on hopanoids [7]. Today it is known that the sesquiterpenes in *Vitis vinifera* are formed from FPP whose biosynthesis relies on both, the DOXP/MEP and the MVA pathway, and that they specifically accumulate in the wax layer of the berry exocarp [8]. The biosynthesis of the diverse sesquiterpene structures in *V. vinifera* is, however, still largely unexplored, especially with respect to the cyclization mechanisms. Studies by Steele et al., Bülow et al., Martin et al. and theoretical studies by Tantillo show the complexity of the various cyclization reactions [9–12].

In order to analyze the biosynthetic pathways of sesquiterpene hydrocarbons in grape berries, a method was developed by us using comprehensive two-dimensional gas chromatography (GC×GC) coupled to a time-of-flight mass spectrometer (TOF–MS) after headspace-solid phase microextraction (HS-SPME).

The comprehensive two-dimensional gas chromatography first demonstrated on an oil sample in 1991 is suitable for the analysis of highly complex samples [13]. Using GC×GC, all analytes of a sample can be separated on two different capillary separation columns of different selectivity, resulting in optimized resolution. Increased resolution makes coelutions less likely and minimizes chromatographic noise. In addition, modulator-controlled focusing effects favor the detection of analytes in the trace range. Furthermore, GC×GC is used to obtain structured chromatograms, i.e., compounds of common structure classes are grouped. The coupling of comprehensive two-dimensional gas chromatography with mass spectrometry as the “third dimension” also provides important information for substance identification. The combination of these advantages makes the GC×GC one of the most powerful separation methods for the chemical analysis of organic compounds in complex matrices [14]. The efficiency of this modern analysis technology has recently been demonstrated using different types of wine in nontarget screenings [15–17].

In our work, we have combined the advantages of multidimensional gas chromatography with those of in vivo labeling. In vivo labeling makes it possible to identify compounds for which authentic standards are not commercially available, which applies to the majority of compounds found in plant foods [18]. We performed feeding experiments on isolated exocarp of freshly harvested grapes (*Vitis vinifera* L.) using the stable isotope-labeled precursors [5,5-^2^H_2_]-1-deoxy-ᴅ-xylulose (*d*_2_-DOX) and [6,6,6-^2^H_3_]-(±)-mevalonolactone (*d*_3_-MVL) to unambiguously identify sesquiterpene hydrocarbons. Based on the obtained mass spectra of the genuine and deuterated compounds, multistage cyclisation reactions in the course of sesquiterpene biosynthesis could be substantiated for the first time for several structural classes in *V. vinifera*.

## Results and Discussion

**Figure 1:**
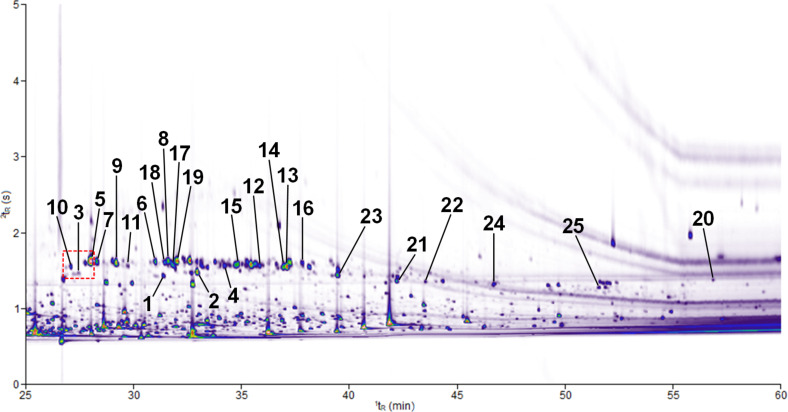
Contour plot of a HS-SPME–GC×GC–TOF–MS chromatogram (TIC) demonstrating the separation of volatile compounds isolated from the headspace of grape berries of the red wine variety Lemberger (*Vitis vinifera* subsp. *vinifera*, clone 1Gm, exocarp). ^1^*t*_R_ (X-axis) corresponds to the retention time on the primary column and ^2^*t*_R_ (Y-axis) to the retention time on the secondary column. The color gradient reflects the intensity of the TOF–MS signal on a white background from low (violet) to high (red). Numbers at peaks refer to compound numbers as defined in Figure 2 and Table 1. The area framed in red including the volatile compounds numbered 10, 3 and 5 is enlarged in Figure 3.

The HS-SPME–GC×GC–TOF–MS analysis of the grape variety Lemberger (*Vitis vinifera* subsp. *vinifera*, clone 1Gm, isolated exocarp) showed the presence of several hundred components. Twenty-five of these compounds were unequivocally identified as sesquiterpene hydrocarbons (Figure 1).

**Figure 2:**
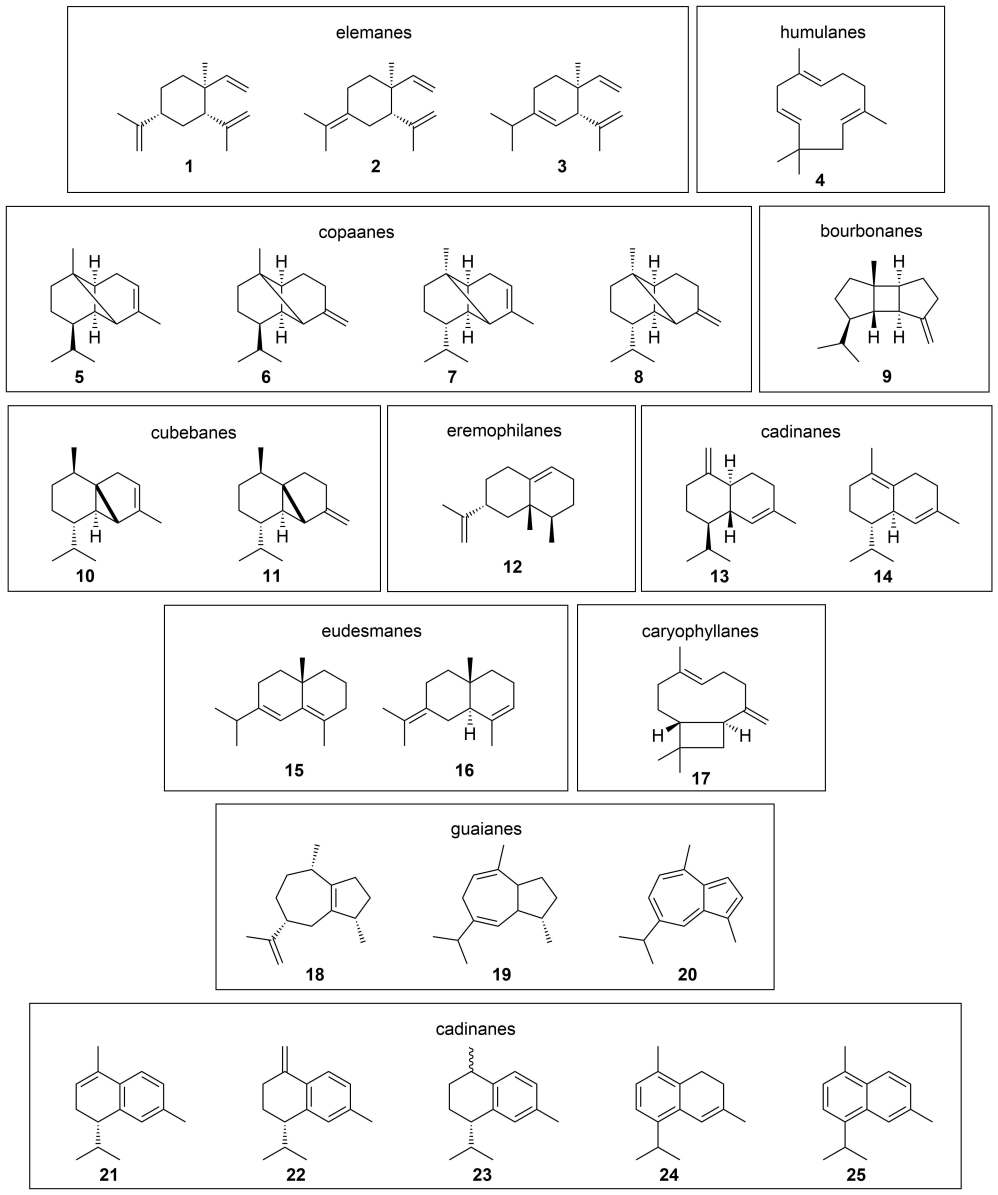
Sesquiterpene hydrocarbons found in the headspace of Lemberger (*Vitis vinifera* subsp. *vinifera*, clone 1Gm, exocarp). **1–4**, monocyclic sesquiterpenes; **5–11**, tricyclic sesquiterpenes; **12–25**, bicyclic sesquiterpenes; **20–25**, aromatic sesquiterpenes. The identified compounds are grouped according to the type of sesquiterpene skeleton.

**Table 1:** Sesquiterpene hydrocarbons identified in headspace of Lemberger (*Vitis vinifera* subsp. *vinifera*, clone 1Gm, exocarp).

compound^a^	*I*^b^	*I* (lit.)^c^	identification^d^/verification^e^

α-cubebene (**10**)	1467	1460 [19]	ms (874, 882), ri, *d*_2_-DOX, *d*_3_-MVL
δ-elemene (**3**)	1478	1479 [20]	ms (859, 864), ri, n.d.^f^, *d*_3_-MVL
α-ylangene (**5**)	1497	1483 [21]	ms (860, 877), ri, *d*_2_-DOX, *d*_3_-MVL
α-copaene (**7**)	1505	1496 [19]	ms (865, 872), ri, *d*_2_-DOX, *d*_3_-MVL
β-bourbonene (**9**)	1532	1519 [19]	ms (853, 863), ri, *d*_2_-DOX, *d*_3_-MVL
β-cubebene (**11**)	1549	1537 [19]	ms (869, 883), ri, *d*_2_-DOX, *d*_3_-MVL
β-ylangene (**6**)	1588	1568 [19]	ms (852, 856), ri, *d*_2_-DOX, *d*_3_-MVL
β-elemene (**1**)	1598	1592 [22]	ms (903, 906), ri, n.d.^f^, *d*_3_-MVL
α-guaiene (**18**)	1600	1591 [23]	ms (871, 892), ri, *d*_2_-DOX, *d*_3_-MVL
β-copaene (**8**)	1605	1598 [24]	ms (847, 856), ri, *d*_2_-DOX, *d*_3_-MVL
(*E*)-β-caryophyllene (**17**)	1612	1604 [25]	ms (928, 939), ri, *d*_2_-DOX, *d*_3_-MVL
guaia-6,9-diene (**19**)	1618	n.a.^g^	ms (824, 833), *d*_2_-DOX, *d*_3_-MVL
γ-elemene (**2**)	1646	1650 [20]	ms (864, 882), ri, *d*_2_-DOX, *d*_3_-MVL
α-humulene (**4**)	1685	1690 [20]	ms (888, 906), ri, *d*_2_-DOX, *d*_3_-MVL
δ-selinene (**15**)	1705	n.a.^g^	ms (841, 887), n.d.^f^, *d*_3_-MVL
(+)-valencene (**12**)	1736	1731 [26]	ms (884, 904), ri, *d*_2_-DOX, std, n.d.^h^
δ-cadinene (**14**)	1769	1770 [20]	ms (858, 900), ri, *d*_2_-DOX, *d*_3_-MVL
γ-cadinene (**13**)	1774	1760 [23]	ms (852, 881), ri, *d*_2_-DOX, *d*_3_-MVL
selina-3,7(11)-diene (**16**)	1796	1778 [27]	ms (872, 881), ri, n.d.^f^, *d*_3_-MVL
calamenene (isomer) (**23**)	1845	1837 [27]	ms (786, 814), ri, n.d.^f^, *d*_3_-MVL
α-calacorene (**21**)	1929	1919 [27]	ms (746, 854), ri, n.d.^f^, *d*_3_-MVL
β-calacorene (**22**)	1971	1939 [25]	ms (836, 898), ri, n.d.^f^, *d*_3_-MVL
α-corocalene (**24**)	2073	n.a.^g^	ms (864, 882), n.d.^f^, *d*_3_-MVL
cadalene (**25**)	2237	2231 [21]	ms (869, 880), ri, n.d.^f^, *d*_3_-MVL
guaiazulene (**20**)	2417	n.a.^g^	ms (872, 880), n.d.^f^, std, *d*_3_-MVL

^a^Unidentified compounds are not listed. ^b^Retention index *I* on a DB-WAX Ultra Inert column. ^c^Retention index data from literature. ^d^Compound identification is based on matching mass spectrum to a library spectrum (ms, match factor and reverse match factor given in brackets, identical mass spectra would produce a match factor of 1000), identical or closely matching retention index (ri) and comparison to a commercially available standard compound (std). ^e^Verification of the found sesquiterpene hydrocarbons was carried out by in vivo labeling with [5,5-^2^H_2_]-1-deoxy-ᴅ-xylulose (*d*_2_-DOX) and [6,6,6-^2^H_3_]-(±)-mevalonolactone (*d*_3_-MVL) as stable isotope-labeled precursors. ^f^The compound could not be detected in *d*_2_-DOX feeding experiments or the mass spectra could not be evaluated. ^g^Retention index data on a WAX column were not available. ^h^The compound could not be detected in *d*_3_-MVL feeding experiments or the mass spectra could not be evaluated.

**Figure 3:**
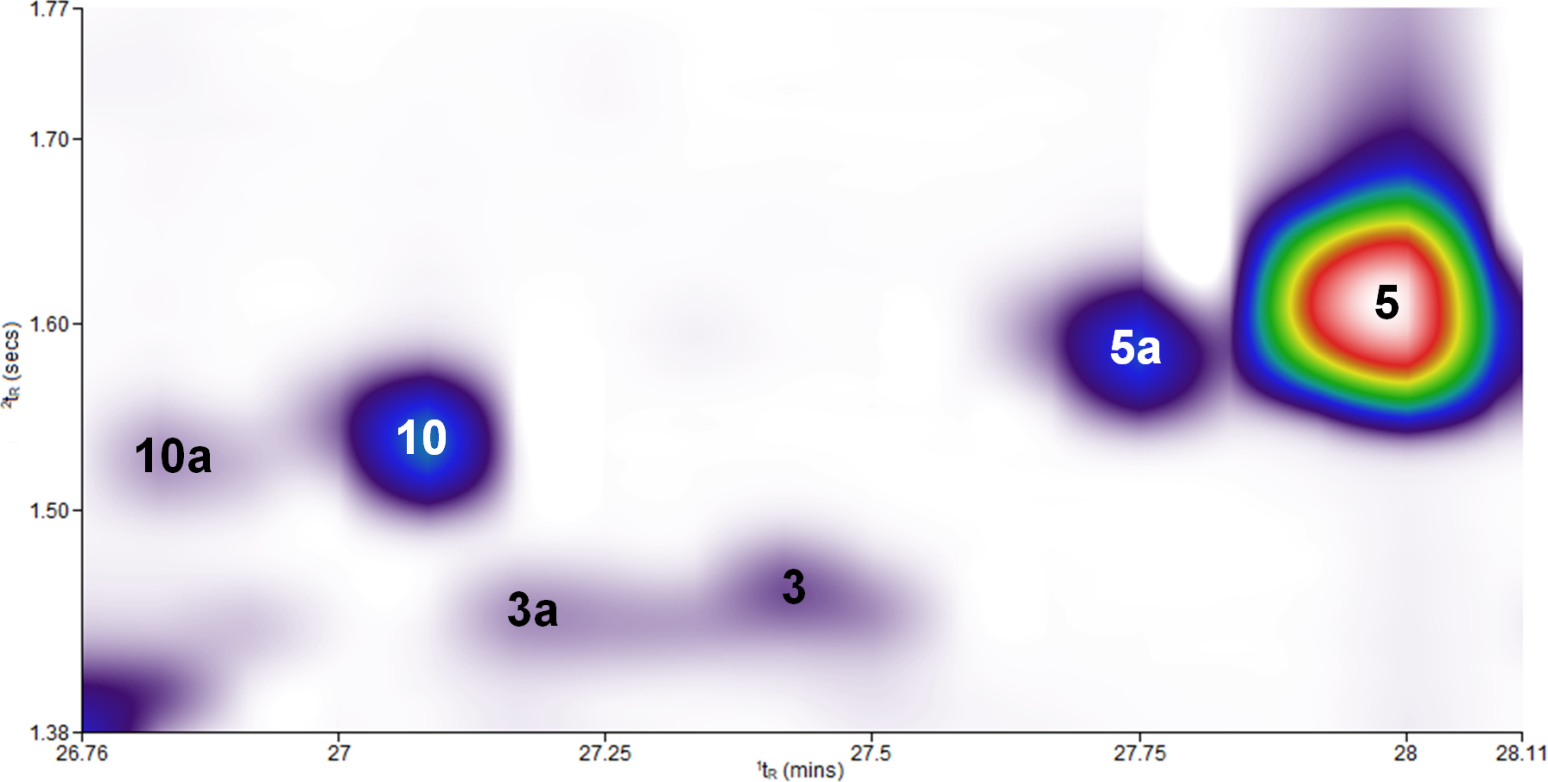
Detailed part of the two-dimensional contour plot (Figure 1) to demonstrate the result of a successful feeding experiment after administration of the stable isotope-labeled precursor [6,6,6-^2^H_3_]-(±)-mevalonolactone (*d*_3_-MVL) to isolated exocarp of grape berries (Lemberger cultivar). The regions labeled with the numbers **10**, **3** and **5** correspond to the genuine sesquiterpene hydrocarbons as defined in Table 1. **10a** (*d*_8_), **3a** (*d*_9_) and **5a** (*d*_8_) are the isotopologues with the highest, possible incorporation of deuterium when *d*_3_-MVL is used as precursor (the maximum possible number of deuterium atoms incorporated is given in brackets). The 3D view of the chromatogram section shown can be found in Supporting Information File 1.

Deuterium-labeled compounds eluted earlier than the unlabeled analogues (Figure 3). This is due to the inverse isotope effect described by Matucha et al. which results in the gas chromatographic separation of isotopologic substances [28]. In the following, the fully deuterium-labeled compounds are examined and their possible biosynthesis pathways described. However, it was also possible to detect the peaks of the partially labeled sesquiterpenes, as Supporting Information File 2 shows using compound **10** as an example.

## Sesquiterpene biosynthesis

### Biosynthesis of sesquiterpene hydrocarbons via (*S*)-(−)-germacrene D

The biosynthetic pathways for the formation of δ-cadinene, α-copaene, β-copaene, α-cubebene and β-cubebene via germacrene D in the legume *Medicago truncatula* have been previously reported by Boland and Garms [29].

**Scheme 1:**
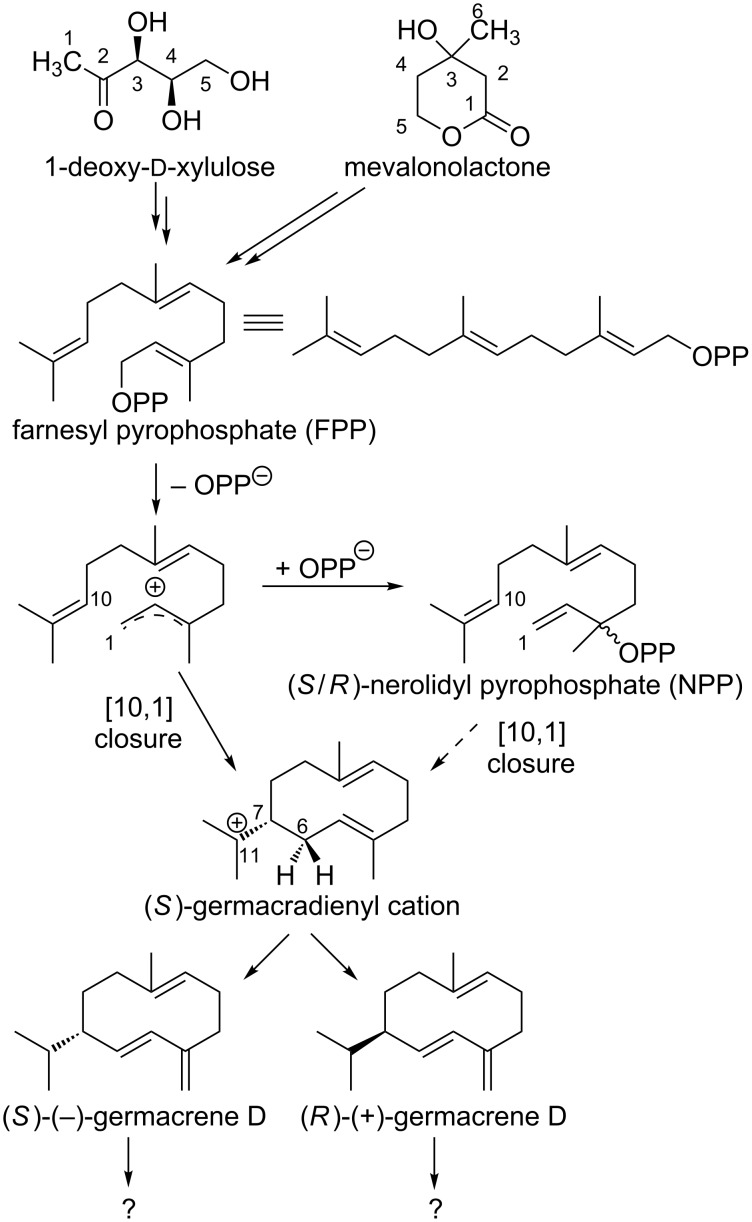
First steps towards the formation of sesquiterpenes. The (*S*)-germacradienyl cation can be formed from FPP or NPP. The subsequent formation of sesquiterpene hydrocarbons via (*S*)- and (*R*)-germacrene D has not yet been clarified.

The formation of the mentioned sesquiterpenes could a priori take place via farnesyl pyrophosphate (FPP) as well as via (*S*)- and (*R*)-nerolidyl pyrophosphate (NPP) (Scheme 1).

Both enantiomers of NPP are possible as intermediates of terpene biosynthesis, since the absolute configurations of their products from *Vitis vinifera* L. are unknown and the subsequent cyclisation reactions can be explained by the enantiomers of germacrene D [30–31].

**Scheme 2:**
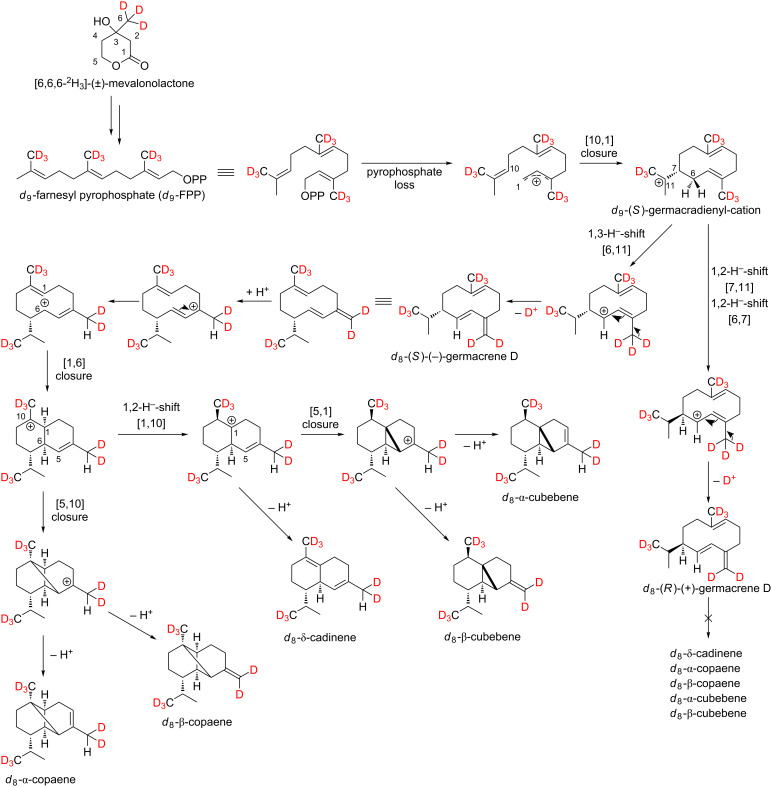
Possible biosynthetic pathways of the sesquiterpene hydrocarbons *d*_8_-α-copaene, *d*_8_-β-copaene, *d*_8_-α-cubebene, *d*_8_-β-cubebene and *d*_8_-δ-cadinene. As a stable isotope-labeled precursor, *d*_3_-MVL was added to isolated exocarp of ripe grapes of the Lemberger grape variety.

In order to investigate whether the formation of δ-cadinene, α-copaene, β-copaene, α-cubebene and β-cubebene occurs via the intermediate farnesyl pyrophosphate (FPP) or via nerolidyl pyrophosphate (NPP), feeding experiments were carried out using the stable isotope-labeled precursor [6,6,6-^2^H_3_]-(±)-mevalonolactone (*d*_3_-MVL, Scheme 2).

As shown in Scheme 2, a maximum incorporation of 8 deuterium atoms was found for the sesquiterpene hydrocarbons, which excludes formation via NPP. Furthermore, these results support the formation of germacrene D, in which the ninth deuterium atom is already missing.

**Scheme 3:**
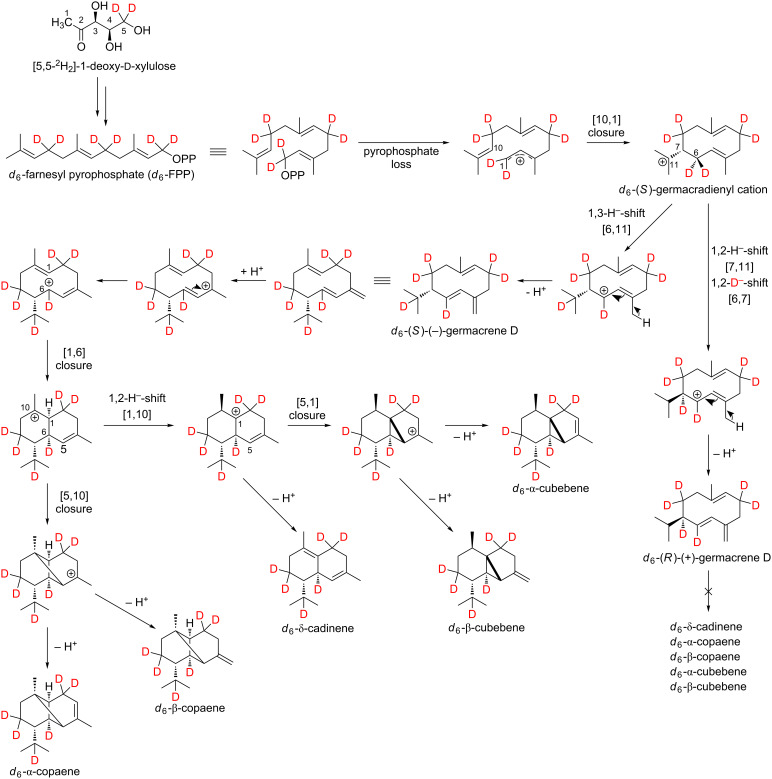
Mechanistic rationale for the generation of the sesquiterpene hydrocarbons δ-cadinene (**14**), α-copaene (**7**), β-copaene (**8**), α-cubebene (**10**) and β-cubebene (**11**) via (*S*)-(−)-germacrene D after feeding isolated exocarp of grapes using the stable isotope-labeled precursor [5,5-^2^H_2_]-1-deoxy-ᴅ-xylulose (*d*_2_-DOX).

Whether the above-mentioned sesquiterpenes are formed via (*S*)-(−)-germacrene D or (*R*)-(+)-germacrene D was investigated by feeding experiments using the precursor [5,5-^2^H_2_]-1-deoxy-ᴅ-xylulose (*d*_2_-DOX, Scheme 3).

Indeed, in Lemberger (*R*)-(+)-germacrene D and (*S*)-(−)-germacrene D are detectable in grape berry exocarp as shown by enantioselective analysis [5,32]. In 1999 Schmidt et al. were able to show that the enantiomers of germacrene D are formed via two different H-transfer pathways in *Solidago canadensis*. (*S*)-(−)-Germacrene D is generated by a cyclization reaction, that includes a 1,3-hydride shift as opposed to the cyclization reaction of (*R*)-(+)-germacrene D, that includes two consecutive 1,2-hydride shifts [33].

In all terpenes in Scheme 3, TOF–EIMS measurements showed that a deuterium atom is located in the isopropyl group. This can be explained by the assumption that in *Vitis vinifera* L. all sesquiterpenes are formed via the (*S*)-germacradienyl cation, which has already been shown for *Solidago canadensis*. The subsequent reactions of the (*S*)-germacradienyl cation to (*S*)-(−)-germacrene D and (*R*)-(+)-germacrene D have already been described above. In the case of the formation of δ-cadinene, α-copaene, β-copaene, α-cubebene and β-cubebene, further cyclization takes place only from (*S*)-(−)-germacrene D, from which the absolute configuration of these substances can be derived.

**Figure 4:**
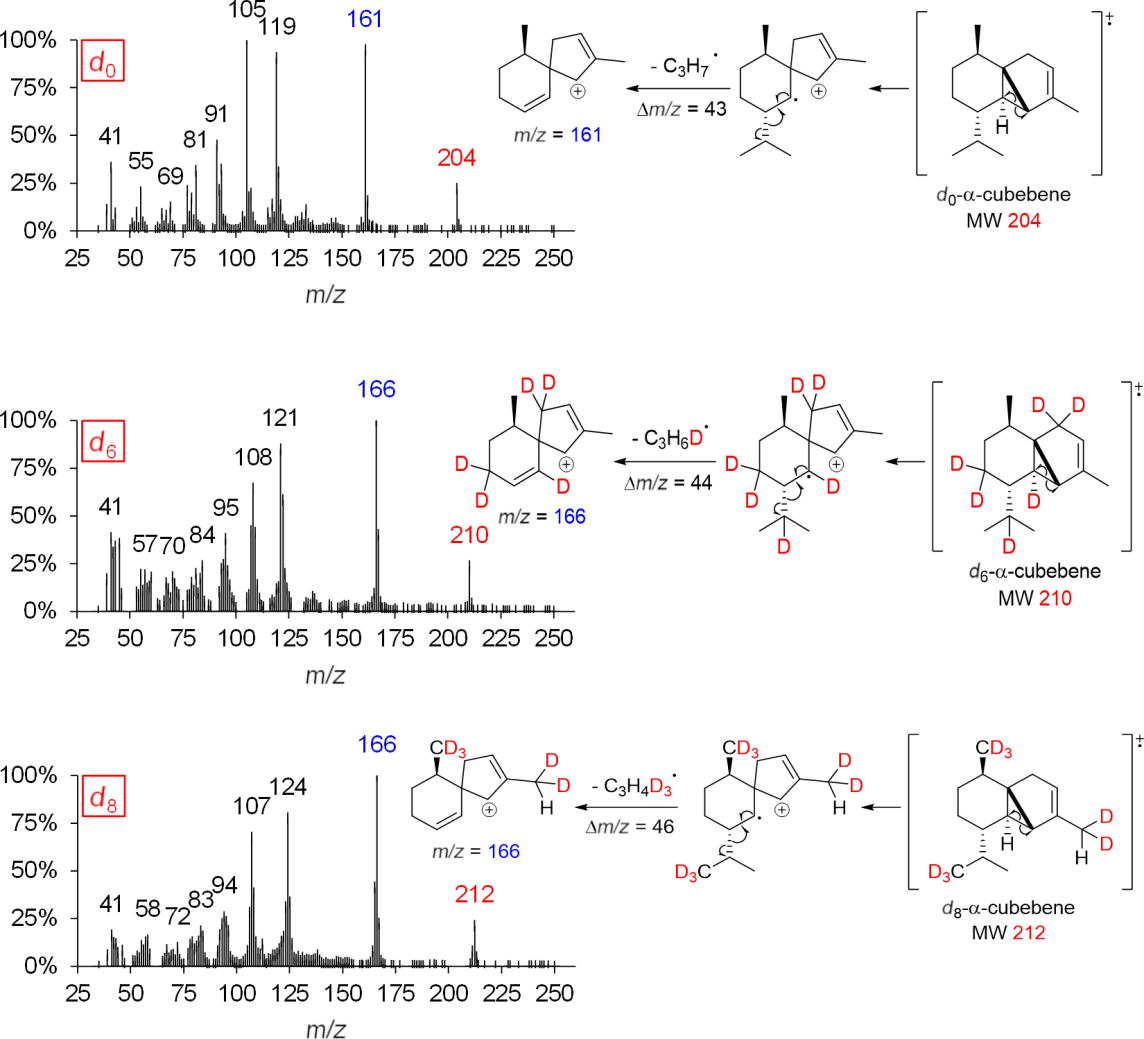
MS spectra of genuine (*d*_0_) and deuterium-labeled (*d*_6_ and *d*_8_) α-cubebene (left panel) after administration of [5,5-^2^H_2_]-1-deoxy-ᴅ-xylulose (*d*_2_-DOX) and [6,6,6-^2^H_3_]-(±)-mevalonolactone (*d*_3_-MVL) to isolated exocarp of grape berries (Lemberger cultivar). Expected labeling patterns are depicted for α-cubebene (right panel).

While the detected labeling patterns in our feeding experiments are in full agreement with the 1,3-hydride shift pathway, the cloning and functional characterization of the corresponding synthase as a final proof is yet missing. In the following, the sesquiterpene hydrocarbon α-cubebene is used as an example to illustrate our interpretations in more detail (Figure 4).

Genuine (*d*_0_) α-cubebene has a molecular ion peak [*M*]^+•^ at *m*/*z* = 204 (Figure 4, upper mass spectrum, marked red). The signal with *m*/*z* = 161 [*M*]^+^ corresponds to the fragment after abstraction of the isopropyl group (marked blue).

Feeding experiments using *d*_2_-DOX showed that according to the biosynthesis pathway demonstrated in Scheme 3, a maximum of 6 deuterium atoms are incorporated into α-cubebene. The presence of *d*_6_-α-cubebene could be confirmed by mass spectrometry as the isotopologue with the highest, possible incorporation of deuterium when *d*_2_-DOX was used as a precursor. The molecular ion peak [*M*]^+•^ of *d*_6_-α-cubebene therefore has a mass-to-charge ratio of 210. The abstraction of a single deuterated isopropyl group leads to the fragment with *m*/*z* = 166. At this point it becomes obvious why it can be assumed that the tricyclic sesquiterpene hydrocarbon α-cubebene is synthesized via (*S*)-(−)-germacrene D and not via (*R*)-(+)-germacrene D. Assuming the biosynthesis of α-cubebene takes place via (*R*)-(+)-germacrene D, the isopropyl group of the molecule would not be deuterated and, therfore, after abstraction of the isopropyl group, the signal with *m*/*z* = 167 would dominate, which is not the case.

In the course of the biosynthesis of α-cubebene after feeding with *d*_3_-MVL as precursor, a deuterium atom is abstracted, resulting in d_8_-α-cubebene [*M*]^+•^ with *m*/*z* = 212 as highly labeled isotopologue (Figure 4, lower mass spectrum).

### Biosynthesis of sesquiterpene hydrocarbons via (*R*)-(+)-germacrene D

**Scheme 4:**
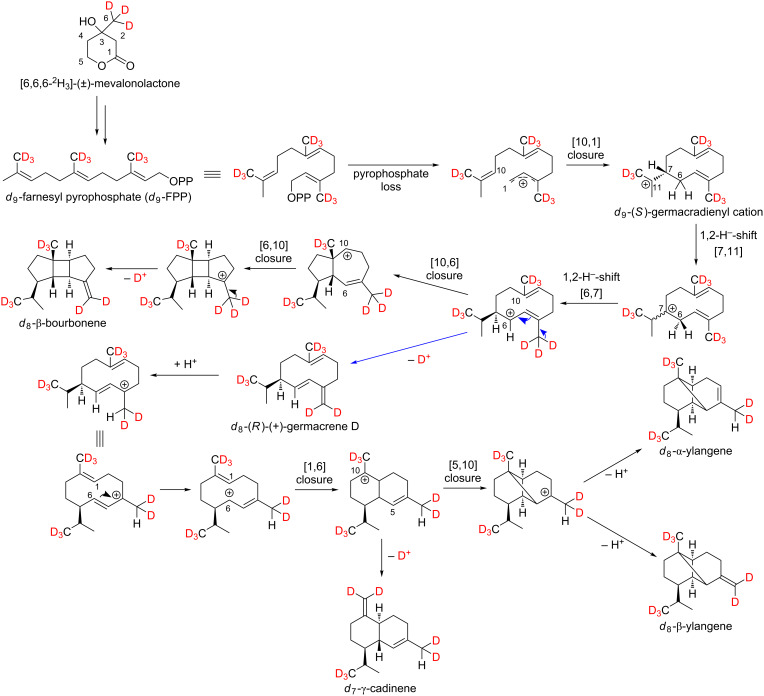
Putative formation pathways of the sesquiterpene hydrocarbons α-ylangene (**5**), β-ylangene (**6**), β-bourbonene (**9**) and γ-cadinene (**13**) via (*R*)-(+)-germacrene D after feeding experiments using [6,6,6-^2^H_3_]-(±)-mevalonolactone (*d*_3_-MVL).

Lodewyk, Gutta and Tantillo postulated an α-ylangene-forming carbocation cascade based on computer calculations [34]. Tantillo and co-workers showed that germacrene D does not have to be involved in the formation of α-ylangene due to carbocation energetics. However, according to our results the formation of this tricyclic sesquiterpene hydrocarbon and its isomer β-ylangene occurs via the intermediate (*R*)-(+)-germacrene D (Scheme 4), which can, indeed, be detected by enantioselective GC in ripening Lemberger berries [5].

**Figure 5:**
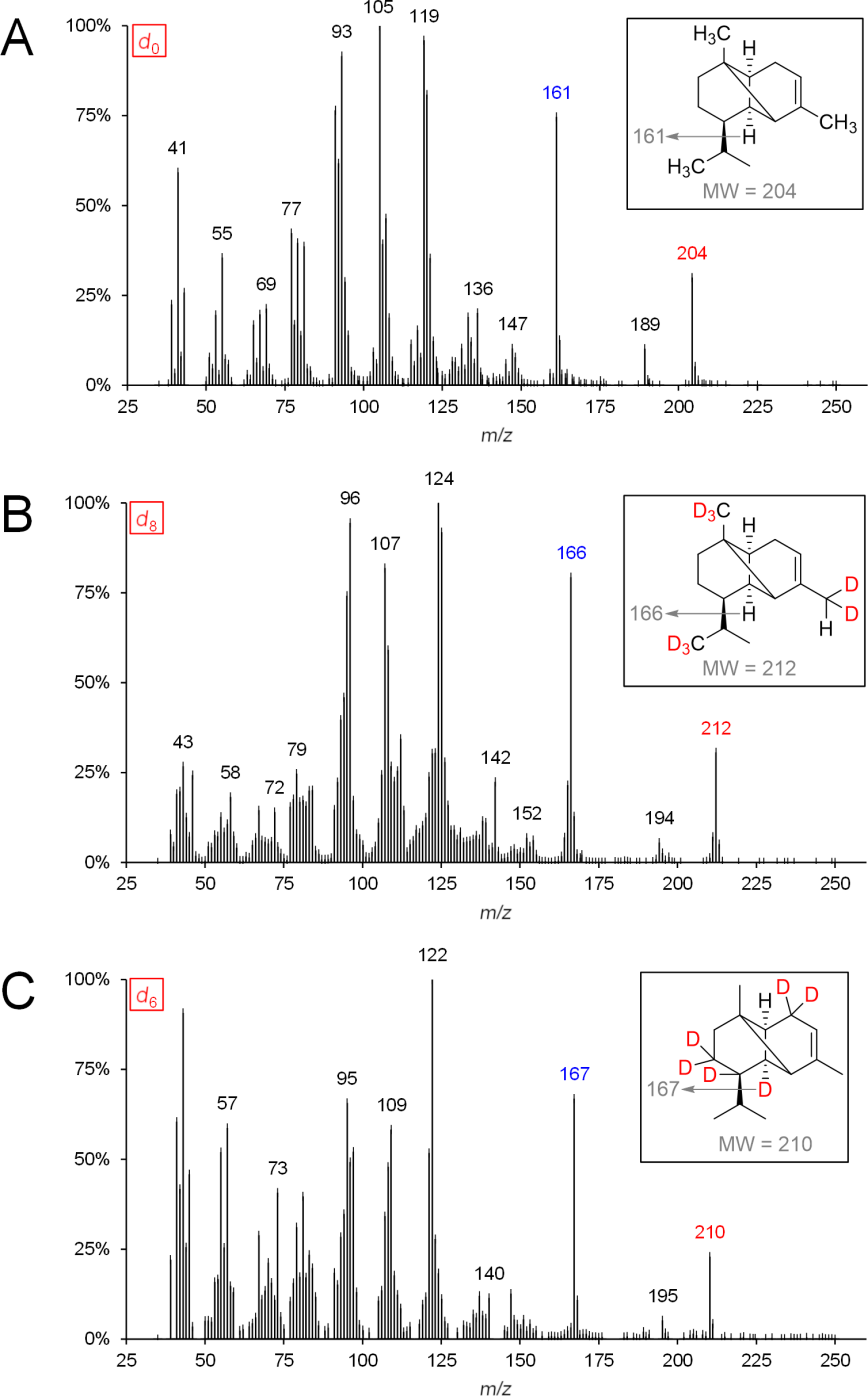
MS spectra and expected labeling patterns of A: *d*_0_-α-ylangene, B: *d*_8_-α-ylangene after administration of *d*_3_-MVL to isolated exocarp of grapes and C: *d*_6_-α-ylangene after feeding experiments using *d*_2_-DOX.

The MS spectra and the expected labeling patterns of genuine (A) and deuterium-labeled *d*_6_-α-ylangene (C) after feeding experiments with *d*_2_-DOX are shown in Figure 5.

Figure 5B shows that after feeding experiments with *d*_3_-MVL a maximum of 8 deuterium atoms are incorporated into α-ylangene, so that a molecular ion peak [*M*]^+•^ with *m*/*z* = 212 is detected. After abstraction of the triple deuterated isopropyl group, the fragment ion is formed with *m*/*z* = 166. According to the computer simulation of Lodewyk, Gutta and Tantillo, *d*_9_-α-ylangene with *m*/*z* = 213 should have been detected after in vivo labeling using [6,6,6-^2^H_3_]-(±)-mevalonolactone (*d*_3_-MVL) as precursor. At this point it should be mentioned that biosynthetic pathways in organisms can differ and computational studies only bear on the intrinsic energetics of possible carbocations.

Furthermore, it is possible that γ-cadinene is formed via (*R*)-(+)-germacrene D. Yoshihara et al. showed that isolated germacrene D reacts to β-bourbonene by irradiation with intense UV light (Hg lamp) [35]. However, the formation of β-bourbonene in plant cells by this photoinduced [2 + 2]-cycloaddition is very unlikely. The biosynthesis of β-bourbonene can be performed by cationic cyclization, as shown in Scheme 4.

**Figure 6:**
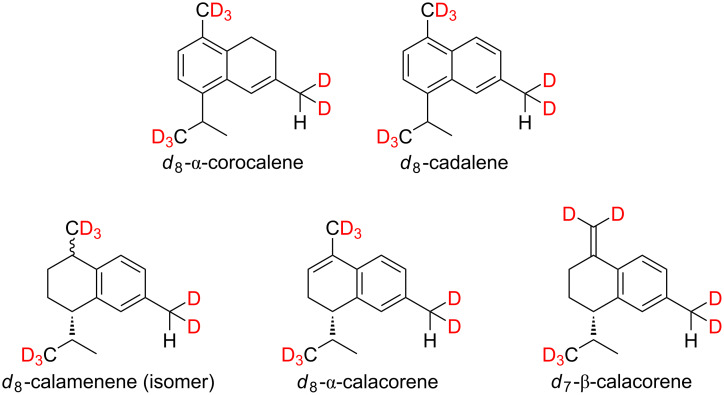
Expected labeling patterns of deuterium-labeled, aromatic sesquiterpenes after administration of [6,6,6-^2^H_3_]-(±)-mevalonolactone (*d*_3_-MVL) to isolated exocarp of grape berries (Lemberger cultivar).

A total of 5 bicyclic, aromatic sesquiterpene hydrocarbons (**21–25**) with a cadinane skeleton were found in exocarp of the grape variety Lemberger, whose identity were confirmed by feeding experiments (Figure 6).

The biosynthesis of the five aromatic sesquiterpene hydrocarbons mentioned is still unknown, but we were able to determine the isotopologues with the highest, possible incorporation of deuterium when *d*_3_-MVL is used as precursor.

**Figure 7:**
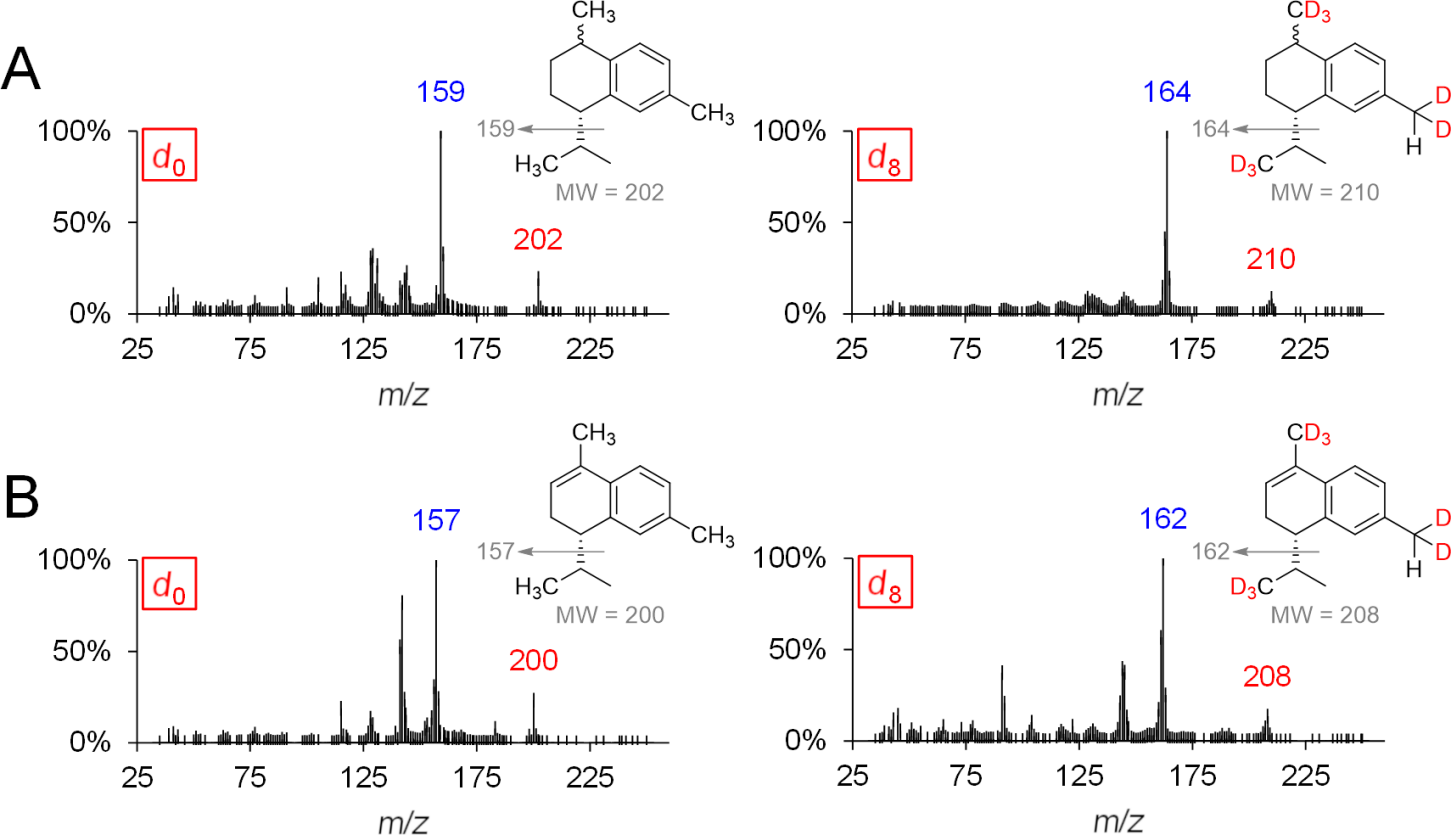
MS spectra and expected labeling patterns of genuine and deuterium-labeled A: calamenene (isomer) and B: α-calacorene after feeding experiments using *d*_3_-MVL.

Figure 7 shows the mass spectrum of genuine (*d*_0_) and deuterium-labeled (*d*_8_) calamenene (isomer) as well as α-calacorene.

### Biosynthesis of sesquiterpene hydrocarbons via germacrene A

**Figure 8:**
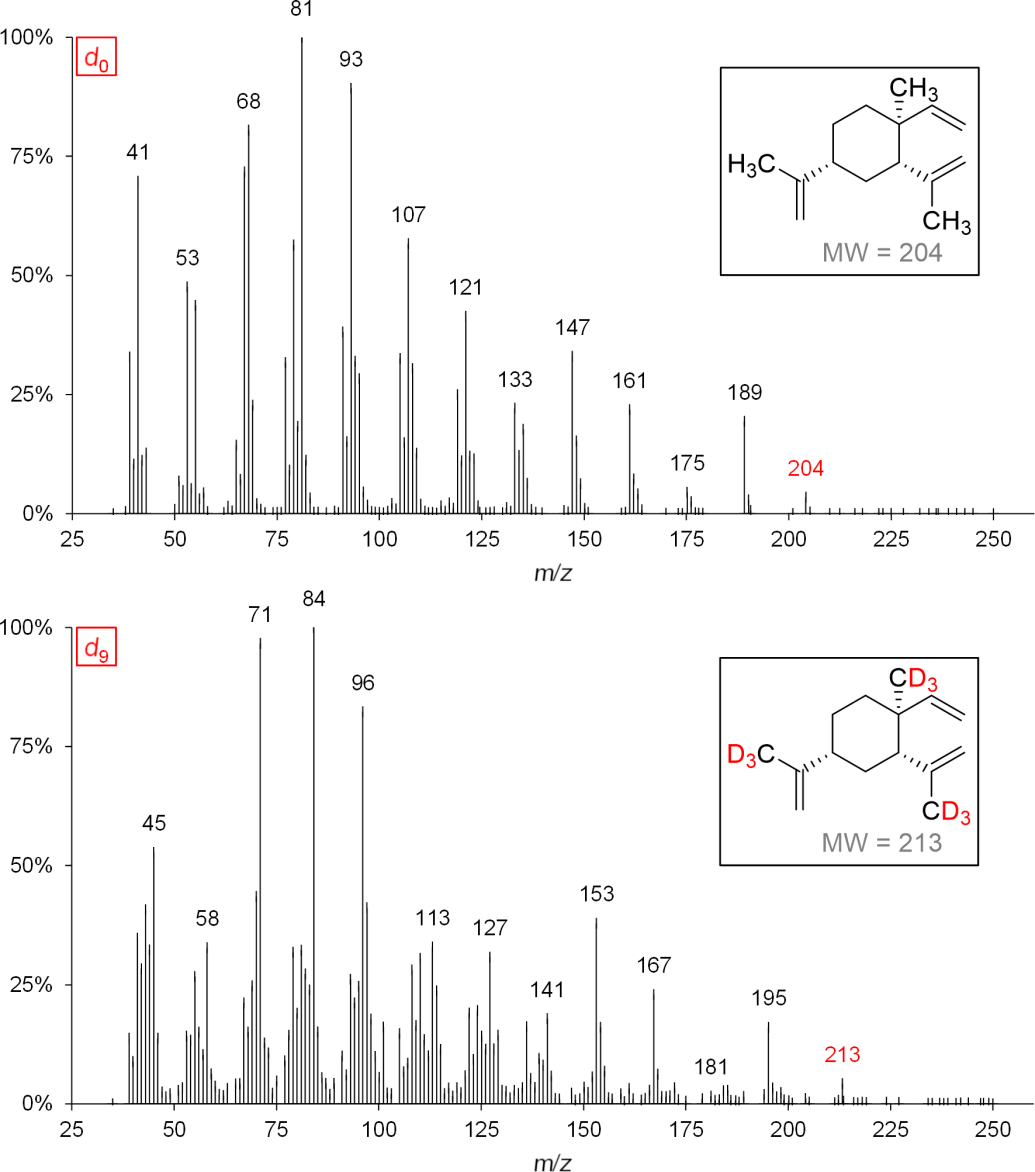
MS spectra and expected labeling patterns of genuine (*d*_0_) and deuterium-labeled (*d*_9_) β-elemene after feeding experiments of grape berry exocarp using *d*_3_-MVL.

**Scheme 5:**
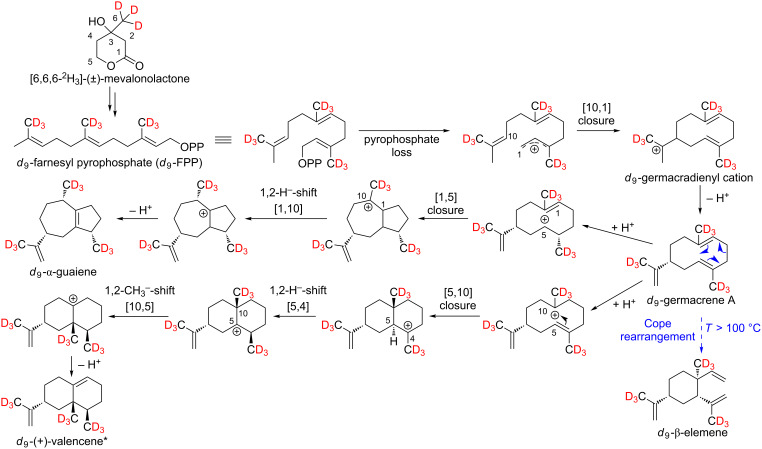
Possible biosynthesis of *d*_9_-β-elemene, *d*_9_-(+)-valencene and *d*_9_-α-guaiene via germacrene A. *An incorporation of deuterium atoms into (+)-valencene could be detected, but due to coeluting substances no characteristic mass spectrum of *d*_9_-(+)-valencene could be obtained.

Faraldos et al. showed that β-elemene is formed by Cope rearrangement when a solution of germacrene A in toluene is heated at reflux [36]. The formation of elemenes requires temperatures >100 °C, so that this reaction cannot take place in the cell, but it must be assumed that these are artifacts of the GC analysis [37]. Nevertheless, elemenes indicate the presence of the corresponding germacrene, as shown by the example of β-elemene and germacrene A (Figure 8 and Scheme 5).

The biosynthesis pathway postulated by Steele et al. for the formation of α-guaiene could also be confirmed [9]. The 2D separation of the sesquiterpene hydrocarbons β-elemene and α-guaiene can be found in Supporting Information File 3. Tantillo suggested the synthesis of (+)-valencene from germacrene A [11]. This biosynthetic pathway could also be confirmed by feeding experiments.

The formation of the three aforementioned sesquiterpene hydrocarbons takes place without deuterium loss, so that with the use of *d*_3_-MVL as precursor (Scheme 5) nine deuterium atoms and with the use of *d*_2_-DOX six deuterium atoms were incorporated at most. This data indicates that the biosynthesis of β-elemene, (+)-valencene and α-guaiene is achieved via germacrene A.

### Biosynthesis of sesquiterpene hydrocarbons via (*E*,*E*)-germacrene B

**Scheme 6:**
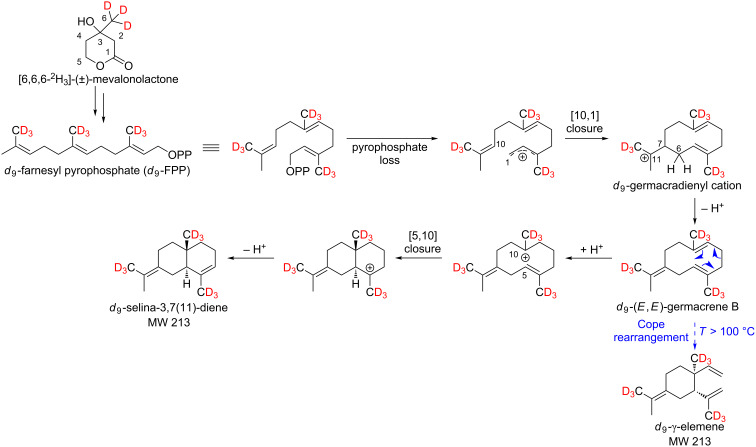
Mechanistic rationale for the generation of the sesquiterpene hydrocarbons γ-elemene and selina-3,7(11)-diene via (*E*,*E*)-germacrene B. The shown deuterium incorporation results from feeding experiments of exocarp (Lemberger cultivar) using the precursor *d*_3_-MVL.

**Figure 9:**
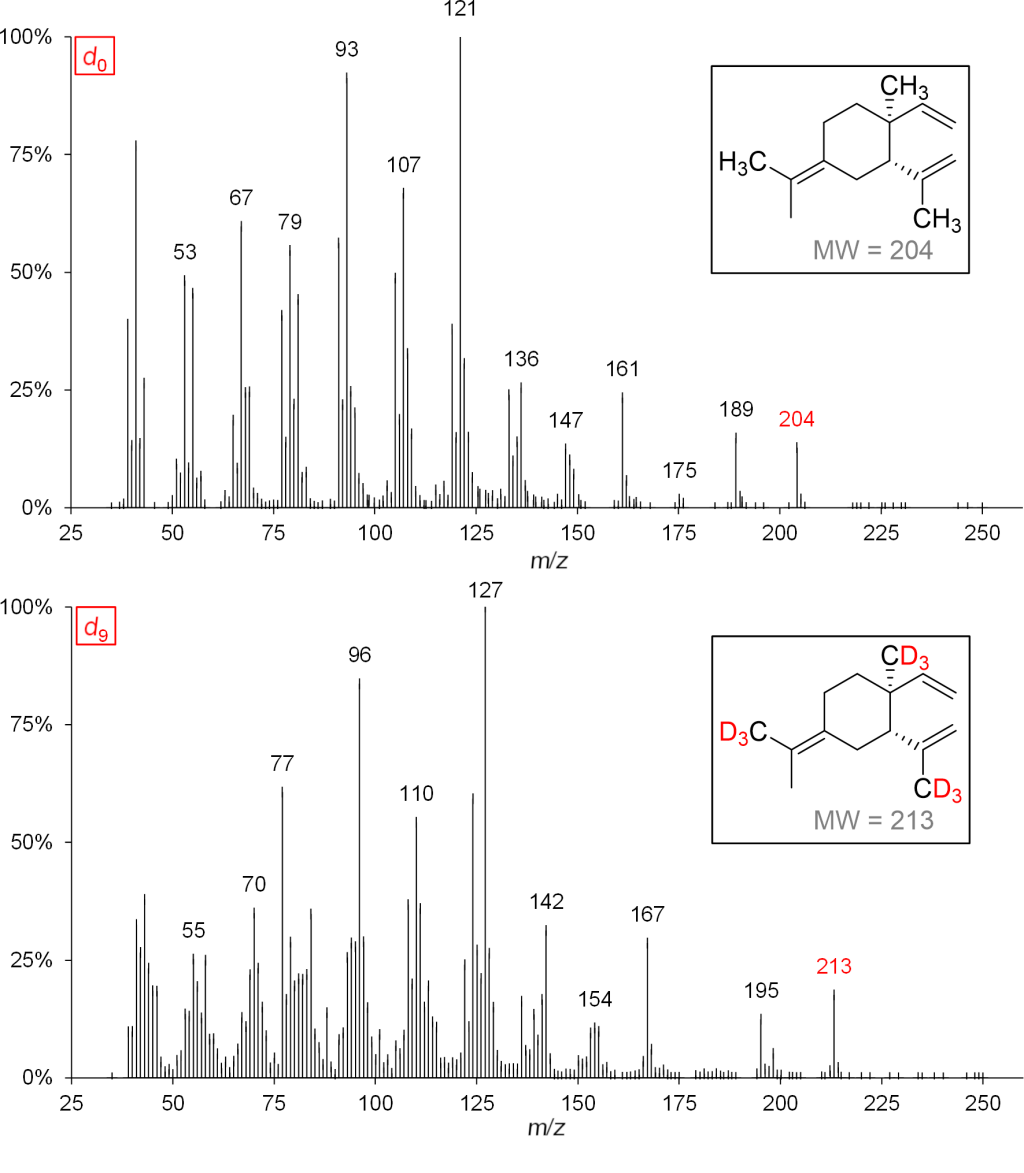
Mass spectra and associated structural formulas of *d*_0_-γ-elemene and *d*_9_-γ-elemene after administration of [6,6,6-^2^H_3_]-(±)-mevalonolactone (*d*_3_-MVL) to isolated exocarp of grape berries (Lemberger cultivar).

The mechanisms proposed by Steele et al. for the formation of selina-3,7(11)-diene and γ-elemene via (*E*,*E*)-germacrene B are consistent with our results (Scheme 6) [9]. In Figure 9 the recorded mass spectra of *d*_0_-γ-elemene and *d*_9_-γ-elemene are shown as examples.

### Biosynthesis of guaiazulene, δ-elemene, guaia-6,9-diene and δ-selinene

**Figure 10:**
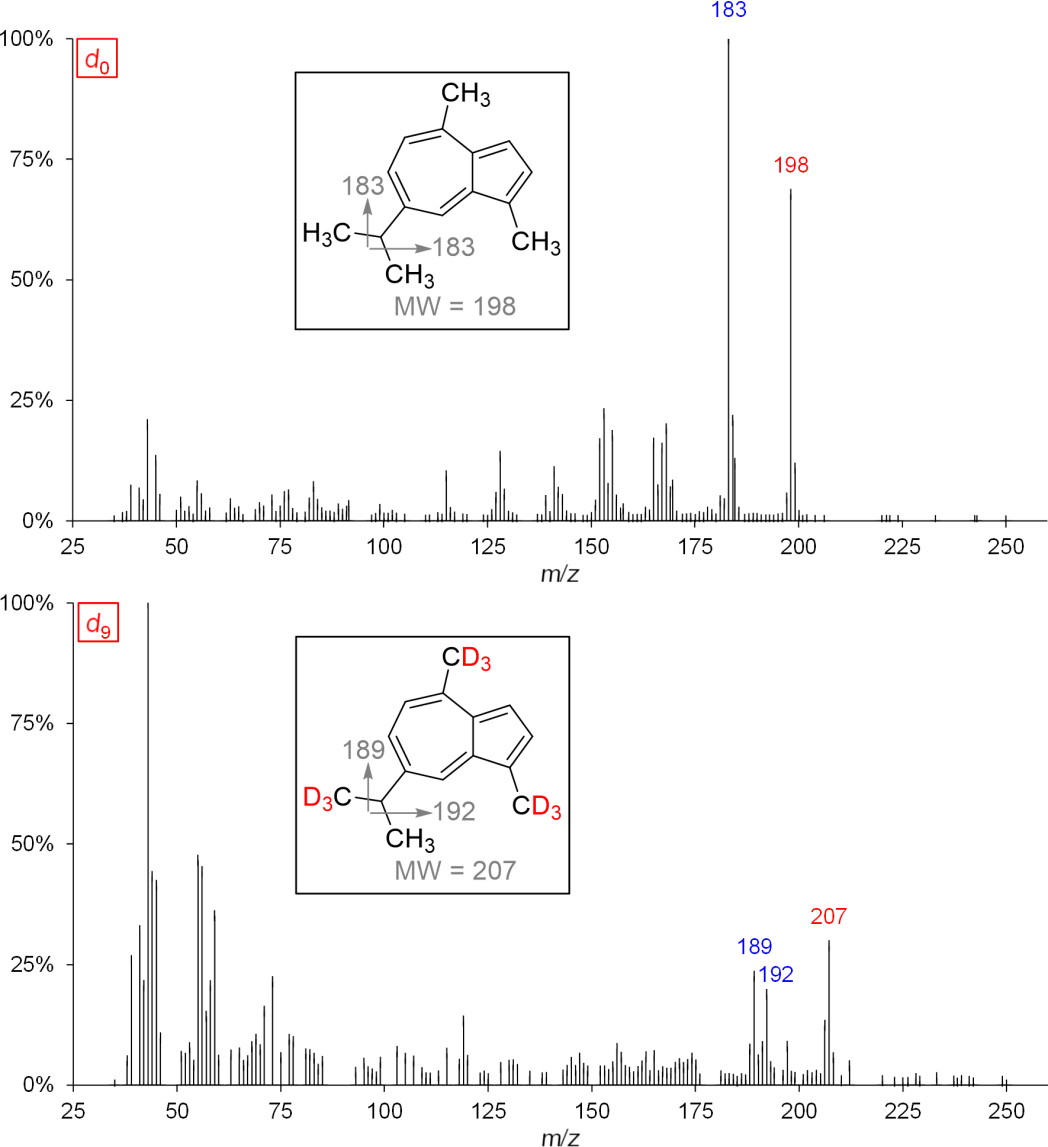
MS spectra and expected labeling patterns of genuine (*d*_0_) and deuterium-labeled (*d*_9_) guaiazulene after feeding experiments of grape berry exocarp using [6,6,6-^2^H_3_]-(±)-mevalonolactone.

The formation of the aromatic sesquiterpene guaiazulene is still unknown. Our feeding experiments with the precursor *d*_3_-MVL show that no deuterium loss is to be expected during the biosynthesis of guaiazulene in grapes, and that a maximum of 9 deuterium atoms are incorporated into guaiazulene (Figure 10).

**Scheme 7:**
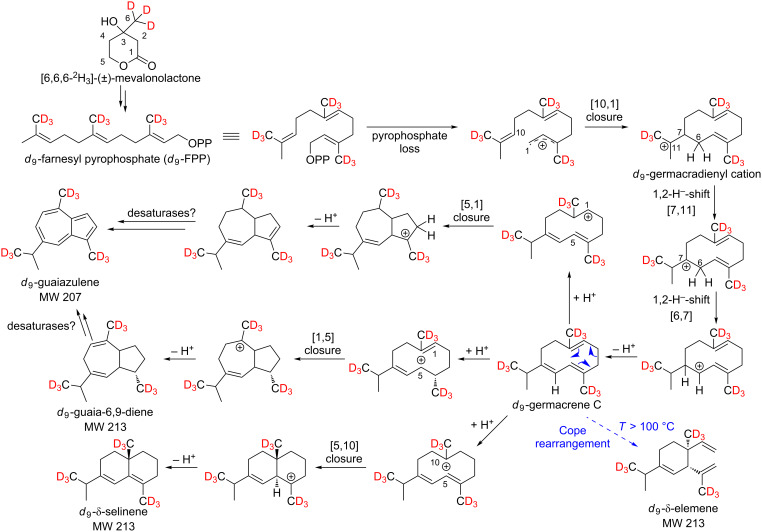
Possible synthesis of *d*_9_-guaiazulene, *d*_9_-δ-elemene, *d*_9_-guaia-6,9-diene and *d*_9_-δ-selinene via germacrene C after administration of [6,6,6-^2^H_3_]-(±)-mevalonolactone (*d*_3_-MVL) to isolated exocarp of grape berries (Lemberger cultivar).

The biosynthesis of guaiazulene could be performed via germacrene C (Scheme 7).

It is conceivable that double bonds are introduced by desaturases in multistage reactions to form the aromatic compound guaiazulene. The participation of desaturases in the formation of aromatic terpenes was already described in 1978 by Poulose et al. explaining the conversion of γ-terpinene to *p*-cymene [38].

May described the formation of δ-elemene from germacrene C via Cope rearrangement [32]. Considering that high temperatures are required for this reaction, our results also indicate that δ-elemene may be formed from germacrene C. The mechanisms described by Steele et al. for the formation of guaia-6,9-diene and δ-selinene via germacrene C could also be confirmed by feeding experiments (Scheme 7) [9].

### Biosynthesis of (*E*)-β-caryophyllene and α-humulene

**Scheme 8:**
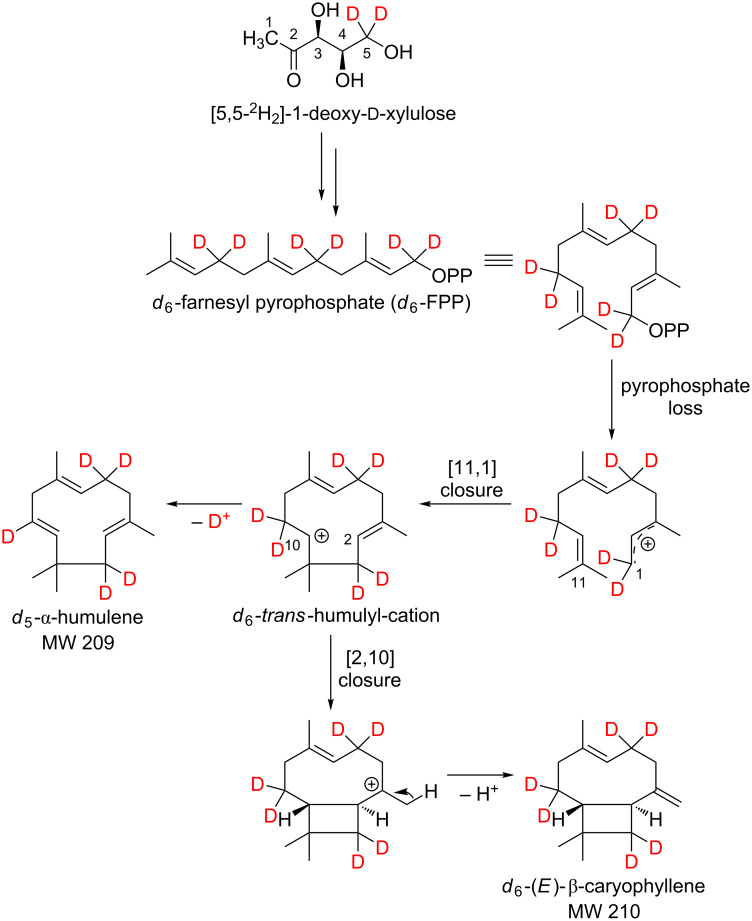
Possible biosynthesis of *d*_6_-(*E*)-β-caryophyllene and *d*_5_-α-humulene starting from farnesyl pyrophosphate (FPP) after administration of [5,5-^2^H_2_]-1-deoxy-ᴅ-xylulose (*d*_2_-DOX) to isolated exocarp of grape berries (Lemberger cultivar).

The biosynthetic pathway postulated by Boland and Garms for the formation of (*E*)-β-caryophyllene from farnesyl pyrophosphate (FPP) is consistent with our feeding experiments (Scheme 8) [29].

**Figure 11:**
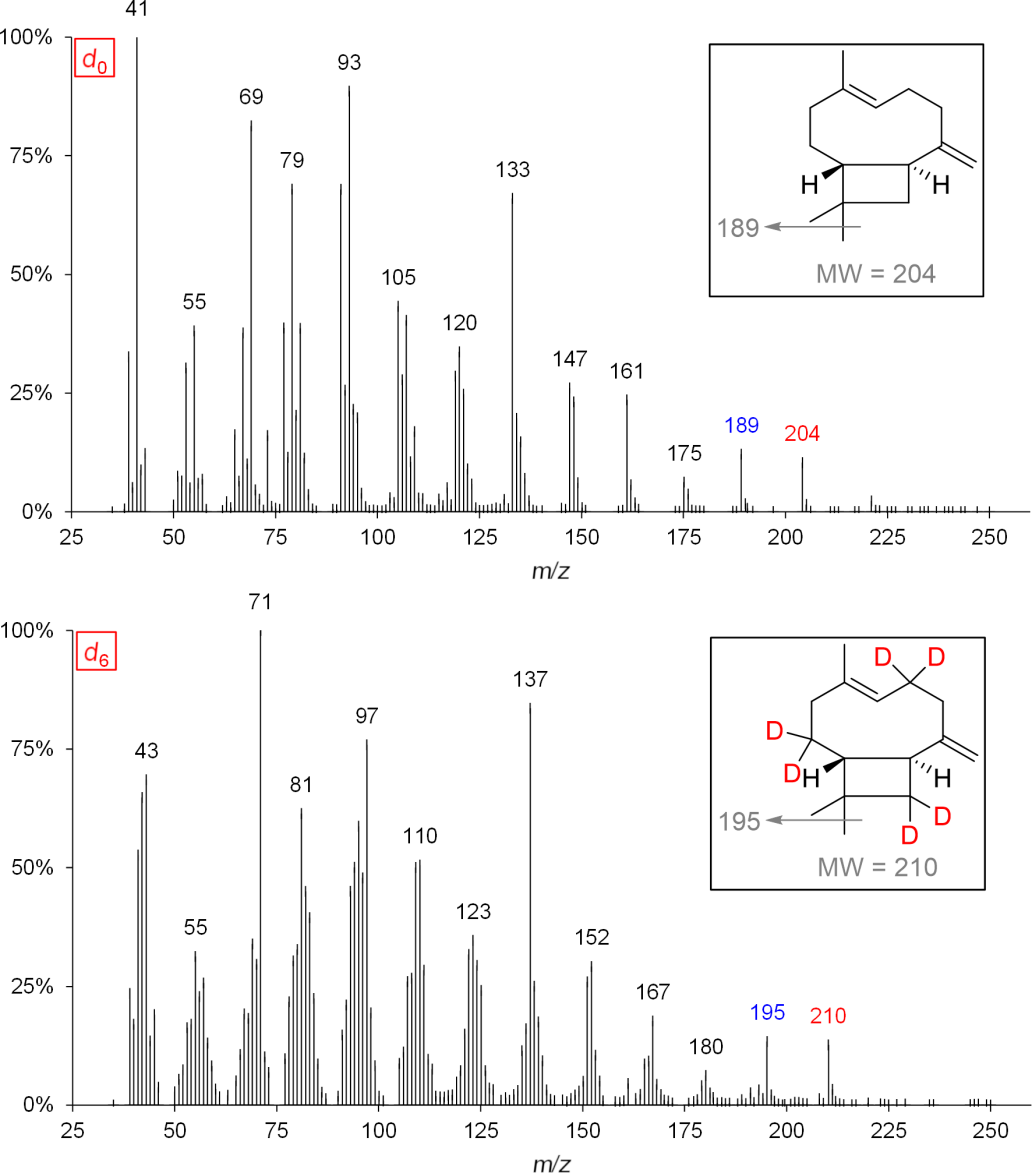
MS spectra and expected labeling patterns of *d*_0_-(*E*)-β-caryophyllene and *d*_6_-(*E*)-β-caryophyllene after successful administration of *d*_2_-DOX to isolated exocarp of grapes of the red wine variety Lemberger.

The incorporation of deuterium atoms into (*E*)-β-caryophyllene during biosynthesis is shown by the following mass spectra (Figure 11).

The assumed biosynthetic pathway for the formation of (*E*)-β-caryophyllene after administration of the precursor [6,6,6-^2^H_3_]-(±)-mevalonolactone (*d*_3_-MVL) can be found in Supporting Information File 4.

Steele et al. described the formation of α-humulene via a *trans*-humulyl cation [9]. This metabolic pathway could also be confirmed by feeding experiments.

## Conclusion

In summary, 25 sesquiterpene hydrocarbons were found in the exocarp of ripe grapes (*Vitis vinifera* L.) of the red wine variety Lemberger. The unequivocal identification of these structurally complex natural compounds was performed by combining multidimensional gas chromatography with in vivo labeling using the stable isotope-labeled precursors [5,5-^2^H_2_]-1-deoxy-ᴅ-xylulose (*d*_2_-DOX) and [6,6,6-^2^H_3_]-(±)-mevalonolactone (*d*_3_-MVL). Based on the recorded mass spectra of genuine sesquiterpene hydrocarbons and their deuterated analogs, the respective labeling patterns were deduced. Subsequently, it was examined whether the multistage cyclization reactions for the formation of sesquiterpene hydrocarbons postulated so far are consistent with our data. Contrary to previous assumptions, it could be shown that the tricyclic compounds α-copaene and β-copaene may be biosynthesized via (*S*)-(−)-germacrene D, whereas their stereoisomers α-ylangene and β-ylangene are formed via (*R*)-(+)-germacrene D. Furthermore, our results showed that the isomeric compounds α-cubebene and β-cubebene are also formed via (*S*)-(−)-germacrene D. In addition, 6 aromatic sesquiterpene hydrocarbons from the headspace of grape berry exocarp of the red wine variety Lemberger were confirmed by in vivo labeling.

## Experimental

### Plant material

Ripe grape berries of the red wine variety Lemberger also called Blauer Limberger (*Vitis vinifera* subsp. *vinifera*, clone 1Gm) from the Hochschule Geisenheim University (Geisenheim, HE, Germany), harvested at the beginning of September 2018, were analyzed. The grapes were transported directly after the harvest to the University of Bonn (Bonn, NW, Germany) for sample preparation.

### Chemicals

The sesquiterpene biosynthesis precursor [5,5-^2^H_2_]-1-deoxy-ᴅ-xylulose (*d*_2_-DOX) was synthesized according to Meyer et al. [39], while [6,6,6-^2^H_3_]-(±)-mevalonolactone (*d*_3_-MVL) was purchased from C/D/N Isotopes Inc. (Pointe-Claire, Quebec, Canada). Retention indices were calculated using a C_7_–C_30_ saturated alkanes standard solution obtained from Sigma-Aldrich (St. Louis, MO, U.S.A.).

The analytical standard (+)-valencene (purity: 80.0%) was also acquired by Sigma-Aldrich (St. Louis, MO, U.S.A.). Guaiazulene (purity: 99.8%) was purchased from Alfa Aesar (Ward Hill, MA, U.S.A.). Water for dilutions was purified using a Milli-Q water purification system (Merck Millipore, Billerica, MA, U.S.A.). Parafilm^®^ M was purchased from Sigma-Aldrich (St. Louis, MO, U.S.A.) to seal the sample vials to prevent the possible evaporation of volatile analytes.

### SPME-Fiber

A divinylbenzene/carboxen/polydimethylsiloxane (DVB/CAR/PDMS, 50/30 µm) SPME fiber from Supelco (Bellefonte, PA, U.S.A.) was used.

### Sample preparation

Sample preparation was based on a slightly modified method described by May, Lange and Wüst [5]. The exocarp of freshly harvested grapes was isolated with a scalpel. One hundred µL of a 0.1% *d*_3_-MVL solution were pipetted into a 10 mL headspace vial and the isolated exocarp was inserted into the solution using tweezers. Similarly, the isolated exocarps of wineberries were placed in 100 µL of a 0.1% *d*_2_-DOX solution. The filled 10 mL headspace vials were closed with screw cap, septum and parafilm. The fed sample material was incubated at room temperature for exactly 48 hours. Subsequently, the samples were stored at −20 °C until measurement. The SPME extraction conditions were based on a slightly modified method described by Welke et al. [17]. Prior to SPME extraction, the samples were held at an incubation temperature of 45 °C for 10 min without stirring. The extraction was carried out at 45 °C for 30 min. After sampling the headspace using a mixed fiber, the volatile and semi-volatile compounds were desorbed in the GC inlet at 250 °C for 5 min. In order to avoid carryover, the fiber was reconditioned for 15 min at 250 °C prior to each analysis. No sample carryover was observed.

### GC×GC–TOF–MS analysis

All chromatographic separations were performed by using an Agilent 7890B gas chromatograph (Agilent Technologies, Palo Alto, CA, U.S.A.) equipped with a liquid nitrogen cryogenic modulator (Consumable-Free ZX2 thermal modulator) coupled to a time-of-flight mass spectrometer (Markes International Ltd, Llantrisant, RCT, UK). Liquid nitrogen was used for cryofocusing of the analytes eluting from the first dimension (1D) column, whereas heated air was used for releasing and reinjecting these compounds onto the second dimension (2D) column. A modulation period of 5 s and a hot jet duration of 0.35 s were used. The column set consisted of a 30 m × 0.25 mm i.d. × 0.25 µm d_f_ DB-WAX Ultra Inert (Agilent Technologies, Bellefonte, PA, U.S.A.) as a high polar primary column with poly(ethylene glycol) as stationary phase coupled to a 1.7 m × 0.10 mm i.d. × 0.10 µm d_f_ MEGA-17 MS FAST (MEGA s.n.c., Legnano, MI, IT) moderately polar second dimension with poly(50%-phenyl-50%-methylsiloxane) as stationary phase. The separation was performed using the following temperature program: 1D oven ramp: 35 °C (5 min), 5 °C min^−1^ to 120 °C (0 min), 3 °C min^−1^ to 220 °C (5 min); 2D oven ramp: 60 °C (5 min), 5 °C min^−1^ to 145 °C (0 min), 3 °C min^−1^ to 245 °C (5 min). The injector was operated at 250 °C in the splitless mode. Helium was used as carrier gas at a constant flow of 1.0 mL min^−1^ and an initial inlet pressure of 166 kPa. The transfer line was maintained at 250 °C and the analytes were ionized by operating in electron impact (EI) mode at −70 eV. The ion source temperature was 250 °C and the detector voltage was set to −2236.8 V. Ions in the mass range 35–250 amu were acquired at a rate of 100 spectra s^−1^. All samples were analyzed in triplicate.

### Data analysis

Data processing was performed using the software ChromSpace (Markes International Ltd, Llantrisant, RCT, UK; version 1.2). First, a dynamic background compensation (dbc) was performed with a peak width of 2 s. The genuine sesquiterpene hydrocarbons were identified using the NIST Mass Spectral Search Program (NIST, Gaithersburg, MD, U.S.A.; version 2.2) by comparing the recorded mass spectra with those of the NIST database (when available), taking into account the match factors and reverse match factors. A series of *n*-alkanes (C_7_–C_30_) were analyzed. The retention times of the measured *n*-alkanes on the first GC column were used to calculate the retention indices *I* according to the method of Van Den Dool and Kratz for temperature-programmed gas chromatography [40]. Subsequently, the calculated retention indices of the identified sesquiterpenes were compared with literature values *I* (lit.). The confirmation of the found sesquiterpenes was done via the corresponding deuterium-labeled compounds starting from the precursors *d*_3_-MVL and *d*_2_-DOX after incubation.

## Supporting Information

File 1 3D view of signals from genuine and deuterated α-cubebene, α-ylangene and δ-elemene after feeding experiments using *d*_3_-MVL.

File 2 Extracted ion chromatograms (EIC) of genuine (*d*_0_), partially labeled (*d*_4_, *d*_6_) and fully deuterium-labeled (*d*_8_) α-cubebene after administration of [6,6,6-^2^H_3_]-(±)-mevalonolactone (*d*_3_-MVL) to isolated exocarp of grape berries (Lemberger cultivar).

File 3 Enlarged 2D chromatogram showing the separation of β-elemene, α-guaiene and β-ylangene.

File 4 Putative formation pathway of *d*_8_-(*E*)-β-caryophyllene and *d*_9_-α-humulene starting from *d*_9_-farnesyl pyrophosphate.
