# Supplementary material for: Analysis of sesquiterpene hydrocarbons in grape berry exocarp (Vitis vinifera L.) using in vivo-labeling and comprehensive two-dimensional gas chromatography–mass spectrometry (GC×GC–MS)
Source: Beilstein J Org Chem. 2019 Aug 14;15:1945–61. doi: 10.3762/bjoc.15.190 (PMC6720654; doi:10.3762/bjoc.15.190)
Supplement: File 1 — 3D view of signals from genuine and deuterated α-cubebene, α-ylangene and δ-elemene after feeding experiments using d3-MVL. [file Beilstein_J_Org_Chem-15-1945-s001.pdf]

BEILSTEIN JOURNAL OF ORGANIC CHEMISTRY

# Supporting Information

for

# Analysis of sesquiterpene hydrocarbons in grape berry exocarp (*Vitis vinifera* L.) using in vivo-labeling and comprehensive two-dimensional gas chromatography–mass spectrometry (GC×GC–MS)

Philipp P. Könen and Matthias Wüst

*Beilstein J. Org. Chem.* **2019,** *15,* 1945–1961. doi:10.3762/bjoc.15.190

# 3D view of signals from genuine and deuterated α-cubebene, α-ylangene and δ-elemene after feeding experiments using $d_3$-MVL

License and Terms: This is a supporting information file under the terms of the Creative Commons Attribution License (http://creativecommons.org/licenses/by/4.0). Please note that the reuse, redistribution and reproduction in particular requires that the authors and source are credited.
The license is subject to the *Beilstein Journal of Organic Chemistry* terms and conditions: (https://www.beilstein-journals.org/bjoc)

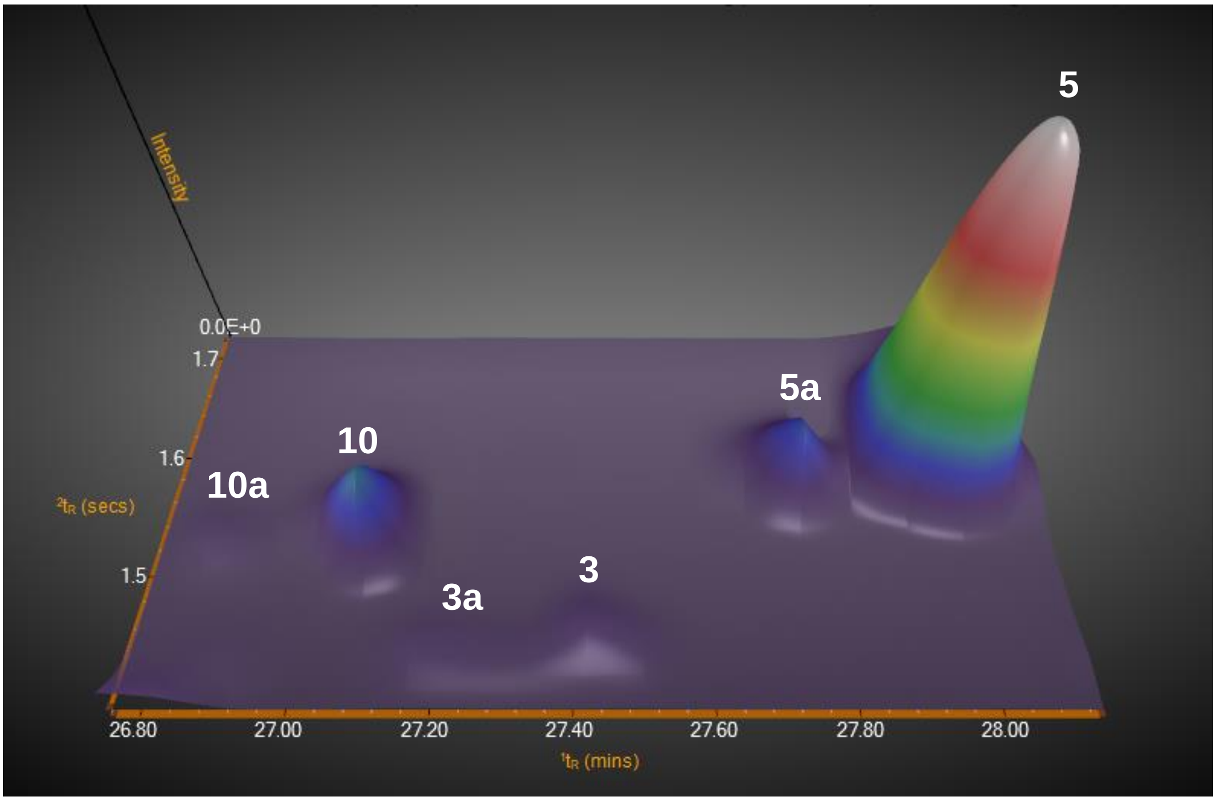


**Figure S1:** Section of a surface chart (TIC) of a HS-SPME–GC×GC–TOF–MS measurement of genuine sesquiterpene hydrocarbons and their deuterated isotopologues after feeding grape berry exocarp of the grape variety Lemberger with $d_3$-MVL. The data is displayed in 3D format, so that the signal intensities are represented in the form of conical signals. Shown are the signals of the two tricyclic sesquiterpene hydrocarbons α-cubebene ($d_0$, **10**; $d_8$, **10a**) and α-ylangene ($d_0$, **5**; $d_8$, **5a**) as well as the monocyclic compound δ-elemene ($d_0$, **3**; $d_9$, **3a**).
