# Supplementary material for: Analysis of sesquiterpene hydrocarbons in grape berry exocarp (Vitis vinifera L.) using in vivo-labeling and comprehensive two-dimensional gas chromatography–mass spectrometry (GC×GC–MS)
Source: Beilstein J Org Chem. 2019 Aug 14;15:1945–61. doi: 10.3762/bjoc.15.190 (PMC6720654; doi:10.3762/bjoc.15.190)
Supplement: File 2 — Extracted ion chromatograms (EIC) of genuine (d0), partially labeled (d4, d6) and fully deuterium-labeled (d8) α-cubebene after administration of [6,6,6-2H3]-(±)-mevalonolactone (d3-MVL) to isolated exocarp of grape berries (Lemberger cultivar). [file Beilstein_J_Org_Chem-15-1945-s002.pdf]

BEILSTEIN JOURNAL OF ORGANIC CHEMISTRY

Supporting Information

for

Analysis of sesquiterpene hydrocarbons in grape berry exocarp (*Vitis vinifera* L.) using in vivo-labeling and comprehensive two-dimensional gas chromatography–mass spectrometry (GC×GC–MS)

Philipp P. Könen and Matthias Wüst

Beilstein J. Org. Chem. **2019,** *15,* 1945–1961. doi:10.3762/bjoc.15.190

Extracted ion chromatograms (EIC) of genuine (d_0), partially labeled (d_4, d_6) and fully deuterium-labeled (d_8) α-cubebene after administration of [6,6,6-$^2H_3$]-(±)-mevalonolactone (d_3-MVL) to isolated exocarp of grape berries (Lemberger cultivar)

License and Terms: This is a supporting information file under the terms of the Creative Commons Attribution License (http://creativecommons.org/licenses/by/4.0). Please note that the reuse, redistribution and reproduction in particular requires that the authors and source are credited.
The license is subject to the *Beilstein Journal of Organic Chemistry* terms and conditions: (https://www.beilstein-journals.org/bjoc)

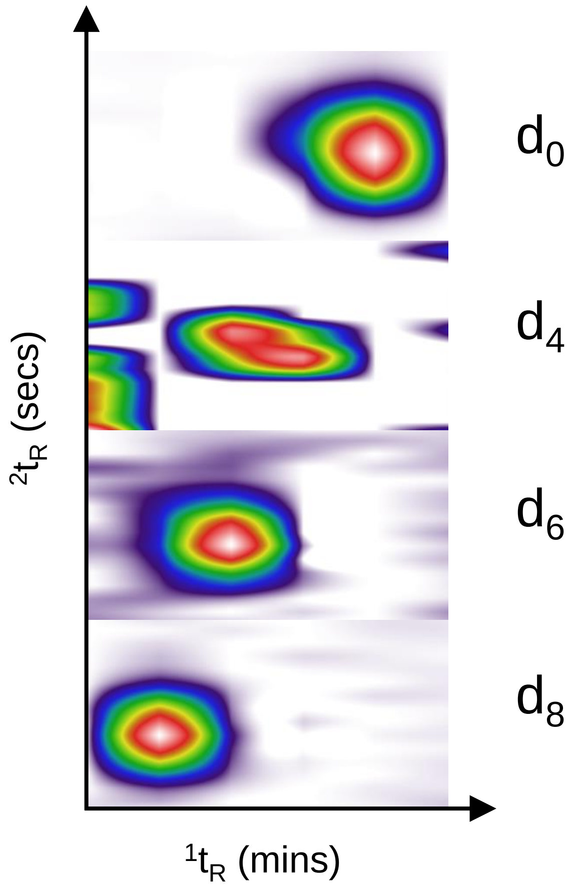

Figure S2: Enlarged sections of a GC×GC chromatogram showing the detected peaks (blobs) of genuine (d_0), partially labeled (d_4, d_6) and fully deuterium-labeled (d_8) α-cubebene after feeding experiments using d_3-MVL. The individual contour plots were obtained by selecting the corresponding mass-to-charge ratios: m/z = 204 (d_0), m/z = 208 (d_4), m/z = 210 (d_6) and m/z = 212 (d_8). The gas chromatographic separation of the shown isotopologues is due to the inverse isotope effect, whereby compounds with higher deuterium incorporation elute earlier.
