# Supplementary material for: Analysis of sesquiterpene hydrocarbons in grape berry exocarp (Vitis vinifera L.) using in vivo-labeling and comprehensive two-dimensional gas chromatography–mass spectrometry (GC×GC–MS)
Source: Beilstein J Org Chem. 2019 Aug 14;15:1945–61. doi: 10.3762/bjoc.15.190 (PMC6720654; doi:10.3762/bjoc.15.190)
Supplement: File 3 — Enlarged 2D chromatogram showing the separation of β-elemene, α-guaiene and β-ylangene. [file Beilstein_J_Org_Chem-15-1945-s003.pdf]

BEILSTEIN JOURNAL OF ORGANIC CHEMISTRY

Supporting Information

for

Analysis of sesquiterpene hydrocarbons in grape berry exocarp (*Vitis vinifera* L.) using in vivo-labeling and comprehensive two-dimensional gas chromatography–mass spectrometry (GC×GC–MS)

Philipp P. Könen and Matthias Wüst

Beilstein J. Org. Chem. **2019,** *15,* 1945–1961. doi:10.3762/bjoc.15.190

Enlarged 2D chromatogram showing the separation of β-elemene, α-guaiene and β-ylangene

License and Terms: This is a supporting information file under the terms of the Creative Commons Attribution License (http://creativecommons.org/licenses/by/4.0). Please note that the reuse, redistribution and reproduction in particular requires that the authors and source are credited.
The license is subject to the *Beilstein Journal of Organic Chemistry* terms and conditions: (https://www.beilstein-journals.org/bjoc)

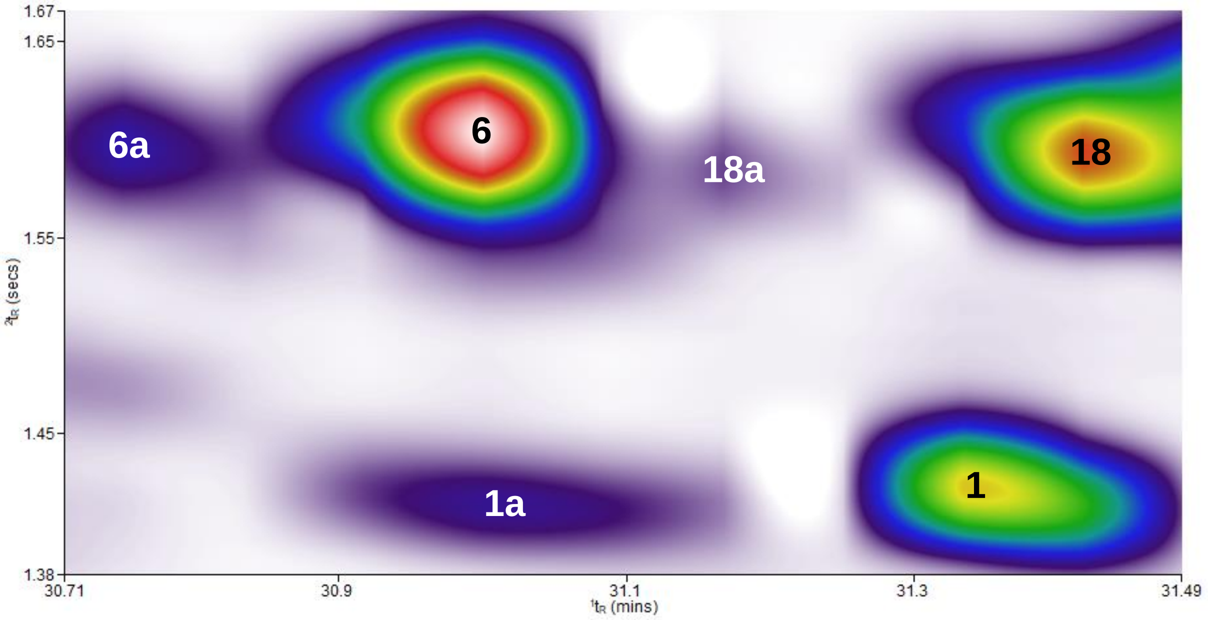

Figure S3: Enlarged, two-dimensional contour plot after successful administration of d_3-MVL to isolated exocarp of grapes of the red wine variety Lemberger. The regions labeled with the numbers **6**, **18** and **1** correspond to the genuine sesquiterpenes β-ylangene, α-guaiene and β-elemene. **6a** (d_8), **18a** (d_9) and **1a** (d_9) are the isotopologues with the highest, possible incorporation of deuterium when d_3-MVL is used as precursor (the maximum possible number of deuterium atoms incorporated is given in brackets).
