# Supplementary material for: Analysis of sesquiterpene hydrocarbons in grape berry exocarp (Vitis vinifera L.) using in vivo-labeling and comprehensive two-dimensional gas chromatography–mass spectrometry (GC×GC–MS)
Source: Beilstein J Org Chem. 2019 Aug 14;15:1945–61. doi: 10.3762/bjoc.15.190 (PMC6720654; doi:10.3762/bjoc.15.190)
Supplement: File 4 — Putative formation pathway of d8-(E)-β-caryophyllene and d9-α-humulene starting from d9-farnesyl pyrophosphate. [file Beilstein_J_Org_Chem-15-1945-s004.pdf]

BEILSTEIN JOURNAL OF ORGANIC CHEMISTRY

Supporting Information

for

Analysis of sesquiterpene hydrocarbons in grape berry exocarp (*Vitis vinifera* L.) using in vivo-labeling and comprehensive two-dimensional gas chromatography–mass spectrometry (GC×GC–MS)

Philipp P. Könen and Matthias Wüst

Beilstein J. Org. Chem. **2019,** *15,* 1945–1961. doi:10.3762/bjoc.15.190

Putative formation pathway of d_8-(*E*)-β-caryophyllene and d_9-α-humulene starting from d_9-farnesyl pyrophosphate

License and Terms: This is a supporting information file under the terms of the Creative Commons Attribution License (http://creativecommons.org/licenses/by/4.0). Please note that the reuse, redistribution and reproduction in particular requires that the authors and source are credited.
The license is subject to the *Beilstein Journal of Organic Chemistry* terms and conditions: (https://www.beilstein-journals.org/bjoc)

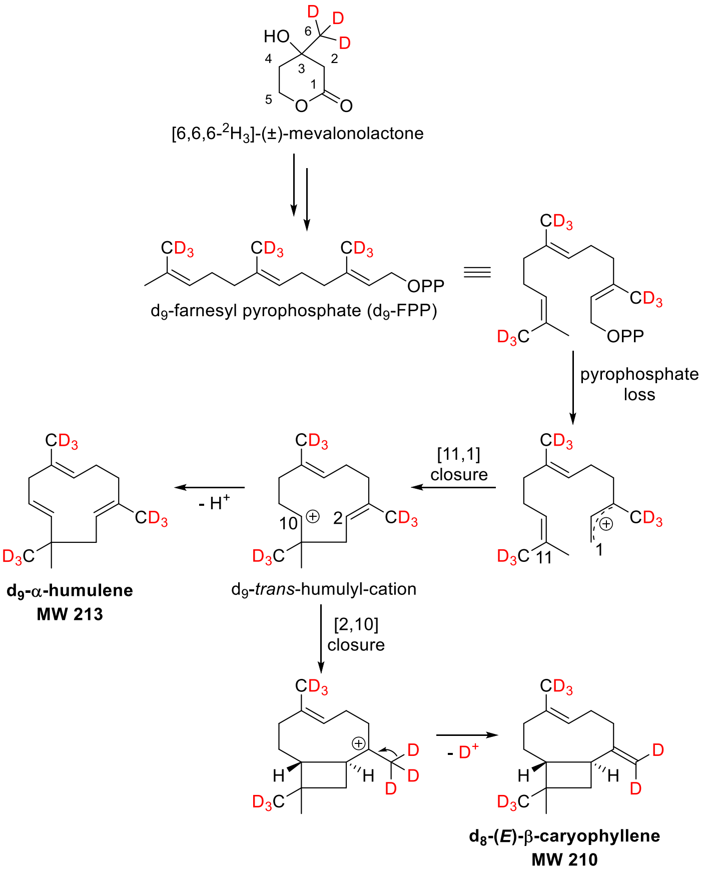

Scheme S1: Possible formation of the sesquiterpene hydrocarbons *d*8-(*E*)-β-caryophyllene and *d*9-α-humulene in grapes from *d*9-farnesyl pyrophosphate (*d*9-FPP). The assumed structural formulas of *d*8-(*E*)-β-caryophyllene and *d*9-α-humulene are based on feeding experiments of isolated exocarp of the grape variety Lemberger using *d*3-MVL.
